# Point of Contact

**Published:** 1897-09

**Authors:** 

**Affiliations:** Lansing


### Point of Contact.

BY DR. MORSE, OF LANSING.

Read before the Michigan State Dental Society.

The object of this paper is to emphasize some established facts familiar to all of you, rather than the presentation of any new or original ideas. Operations for the preservation of the teeth are the principal requirements of the dentist of to-day, and filling teeth is the principal means of their preservation; so that anything that tends to lengthen the durability of fillings is of great importance to both operator and patient. The principles that I am about to present fulfill this mission, I believe, thus making a sufficient apology for consuming your time. The normal set of teeth in the mouth of a human adult number sixty-four, offering sixty points of contact which represents about three-fifths of the probable points at which caries attack the teeth. Why is it that the teeth decay more at this point than others? It is due to the fact that at this point and just away from it toward the cervical margin, the surface is not self-cleansing, and there becomes attached to the teeth a thin film of agglutinated substance, made up principally of micro-organisms. The food in process of mastication does not disturb them. The brush passes over the buccal and lingual surfaces, and still they remain. The tooth-pick will dislodge some, but it is necessary to use dental floss, or something of that nature to polish and scour them off. If all surfaces of the teeth were kept cleaned of this accumulation, we would soon find that our profession was considerably crowded, but the laity have not been sufficiently drilled on this method, and those who have been instructed, do not find the time to do it thoroughly, hence we are called upon to correct the results of their defiance of providence. This agglutinated mass of organisms secrets acids; it first attacks the cement that binds the enamel rods together, on account of its not being as dense as the enamel rods. Then the rods themselves are decalcified, and gradually disintegrated, leaving an opening into the surface. In such openings as fast as found, the microbes establish themselves, and from now on have it all their own way unless the cavities are scientifically stopped. It is our duty to our patients to instruct them in the use of dental floss and brush to thoroughly cleanse the teeth. Abrasion is the only means by which this can safely be accomplished, and then it is necessary that it should be attended to religiously. Mouth-washes aid in making the microbes uncomfortable but do not remove them. Careful brushing and the use of dental floss in connection with mouth-washes is the best method of attack.

But the thing that I wish to call attention to particularly is the restoration of this point of contact by filling. Dr. Black in his talk before the Chicago society, in February, tells of the new method of cutting away the proximal aspects of the molars and bicuspids that are affected by caries, and replacing with large metallic surfaces properly shaped to control the proximal space, and thus offering a roost for the microbes that they cannot destroy with their acids. I quite agree with the doctor in the main, on the counts he has made, in the case of considerable destruction of the enamel and dentine at this point, but I feel that there is a class of cavities that can be treated in a less heroic manner with good results. The moderate and small-sized cavities around which the enamel is not affected would be of this type. Take two moderate sized proximal cavities as an example, in teeth of normal shape and position. At first examination if time permits place between these teeth a pellet of cotton saturated with chloro-percha. This is repeated at intervals of three or four days, increasing the size of the pellet until the space is sufficiently large to permit of *thorough* preparation of cavities and of filling and finishing the fillings, and when the teeth resume their natural position the oval surfaces of the fillings should come in contact, and at no point should the enamel on either tooth touch the opposing filling or tooth. The point of contact should be as near the occlusal surface as consistent with the case. Treated in this manner, the margins of the enamel are as self-cleansing as under the best conditions that nature provides and gives the gums in the proximal space good protection. Drs. Miller and Black have for some time maintained that the fluids of the mouth were responsible for carious teeth, only as they aided or facilitated the growth of organisms. Dr. Williams has demonstrated this to be the fact, and accounts in this way for predisposition to decay, whether the teeth are what are known as hard or soft teeth. In cases of marked predisposition it is probably necessary to apply the method Dr. Black advocates. With incisive teeth we see many cases of re-occurrance of decay at the cervical and at the incisive margin of cavities, that were well prepared and carefully filled, but when they resumed their natural position the point of contact was at either of the margins of the enamel rather than the filling. This result probably was due to not having sufficient space by separating to contour the filling sufficiently to allow of their being finished, and still have the contour to project beyond the margins and act as a buffer to hold the enamel apart, so that they may be cleansed and cleanse themselves to a degree.

I wish to appeal from the decision and practice, of some good men in the profession that the use of the matrix, is in opposition to these results unless an extreme amount of separation has been procured, while the making and the placing of the filling is a little easier. The completed filling does not often meet the requirement of the restoration of the original tooth.

In crown work where the space is contracted, there is a liability to having straight surfaces in contact, it is desirable to avoid this, as separating will often correct the condition so that cleansing spaces will remain and the gum septum be restored.

In conclusion I wish to appeal to the dental profession to be more thorough in separating teeth to be filled, no matter what filling material is to be used. Make the point of contact of a shape that would best protect the proximal space from the encroachment of food. Have the fillings rest against each other holding the enamel apart from touching the adjoining tooth or fillings. This metallic point of contact acts as a protection to the tooth from re-decay as a sled runner is protected from wear by the iron shoe fastened upon it.

DISCUSSION.

DR. SWEETNAM: Dr. Morse has certainly struck an idea of most practical importance. Where the Doctor has placed the most importance is at the point of contact. Let us keep in mind the inter-approximate space. I don't care what the shape of the filling is if that space is preserved. Dr. Black has become a crank on that idea. He brought it out in 1890. In 1892, Dr. Ottolengui, of New York, brought up similar ideas, Dr. Williams, of England, goes on and explains how caries will come in and attack that surface. In regard to securing that space, we have a great many different methods of separating the teeth. There are cases where slow process is necessary. Where decay has been so slow that one tooth has dropped into the cavity of the other, and the inter-proximate space has entirely disappeared. In that case it is necessary to wedge by slow means to recover the lost space. I think it is the only circumstance in which a man is at all excusable for using wedges of any kind. There are many accidents from wedges, where they are driven down and destroy the gum septum. With the bar separator, you can separate the teeth with little or no pain. You don't need a great deal of space, but enough to make a sufficient contour to that filling to preserve the inter-proximate space. The same rule applies to crown work. The point of contact is not of so much consequence so long as the inter-proximate space is preserved. If that isn't regarded, that filling will invariably fail.

I don't care if fillings were put in yesterday, and that has been disregarded, I would take them out at once, and that patient would be made comfortable. It is unnecessary to wait for decay. I would leave a sufficiently strong wall, where, if they let them decay, they get farther than I want them to go. I think a man is justified in removing fillings of that kind. I use the double bar separator. It is necessary to note the space and when those teeth come together, to see that they strike at the point you expect. It is not always necessary to have the teeth strike where they did, but where they ought.

Dr. Morse: If the point of contact, as I have tried to designate, is made with a good understanding of it, it will accomplish the rest at the same time. I have a set of separators: I formerly used them altogether, but I find that in treating teeth where I use cotton pellets and gutta-percha, in many instances I obtain separation without using the separators. I now make a practice of doing so all the time, and find that by making gradual application it is not necessary to make the teeth sore, and by getting sufficient space, when ready to operate, I don't need to have any separators in the way. It doesn't require a great while to do it; ordinarily a week's time is sufficient.

Dr. Hoff: The matter of restoring the contour of teeth and retaining the inter-proximate space is an old one suggested years ago. As long ago as when I came into the profession this was an important subject. Dr. Black seems to be enjoying the reputation of having discovered what was known and talked about as long ago as when cohesive gold fillings were first used. We are greatly indebted to Dr. Black, in behalf of the younger members of the profession, for the scientific way he has stated this important principle. I have always made my fillings on the contour plan. Have never had any sympathy with the practice of separating the teeth. I have always avoided making fillings of that character. I have always made an effort to have as little contact as possible with the fillings. In the beginning I had no other idea of the value of this matter than the esthetic. The knowledge that we now have of bacteriological influence was not known. We said the acid, where left to remain on the side of the teeth by capillary attraction, dissolved the tooth at the joint and it decayed along the border. I was early instructed in the importance of making the borders of the cavities of these fillings secure. I think in these cavities we are inclined to try to preserve too much of the tooth tissue at the cervical border, because of the difficulty in making a perfect joint, and it is difficult to keep dry. I think more fillings fail near the cervical margin than elsewhere. Fillings seldom fail on the lateral border unless from bungling work. The difficulty is not because we fail to get a firm anchorage or finished joint, but we expose a margin peculiarly susceptible to the agents of decay in the mouth. The necessity of making a true border and tight joint is equally important with making the point of contact and preserving the inter-proximate space. I agree heartily with the idea of making contour fillings and maintaining the inter-proximate space. Nothing is more disagreeable than two flat surfaces of teeth together. When we have chicken for dinner, we have to devote the rest of the afternoon to removing the shreds.

Dr. Sweetnam: Dr. Hoff speaks as though this subject of the inter-proximate space was an old one; but it is not, I have been since 1879 a student and knew nothing about the inter-proximate space before Dr. Black came out in 1890, excepting from my own experience. If anyone has written anything on the idea of the retention of the inter-proximate space before 1890, I would like to know it. I also disagree with him in regard to fillings failing mostly at the cervical margin. It is at the *lingual* margin.

Dr. Young: I think I can correct Dr. Sweetnam in his statement that there has been nothing published previous to 1890. Marshall Webb's Notes on Operative Dentistry carry out this idea right through.

Dr. Lowry, of Kansas City: The idea of contouring approximate fillings antedates the idea of giving teeth that saw-tooth form, separating every tooth, making every tooth smaller at the cutting edge than at any other portion. That was a fad and lasted only a short time. The idea of contour approximate fillings is a self-evident fact. We know that if you give fillings a tooth contact instead of a metal contact, that decay will recur. You must to have the margins so formed and situated that friction of food will keep them clean. With reference to the cervical border, I find it is the easiest point to preserve. I will tell you my reason. In the first place you have more direct action upon it with the instruments. You can spread the gold more thoroughly at that point. The failure of fillings at the cervical border is largely due to defective excavation and defective finishing. I give my gold fillings a gold contact. I bring the gold just beyond the point where there would be danger from the tooth substance coming in contact. You can do that in a great majority of cases without any separation at all. Separating is seldom necessary where teeth are partly decayed. In that case you can do pretty well if you have an artistic eye and mind. But give the approximate fillings as nearly as possible a point of contact at the cutting edge, and you won't make a space that will retain food. With a matrix, I claim it is an absolute impossibility to produce a perfectly moisture proof filling margin. Never with a gold filling. It is an invention to save trouble, but not to save teeth. I have never seen a good gold filling put in with a matrix.

Dr. Field: I understood the last speaker to say he considered the cervical margin to be the most invulnerable to decay. I consider it the most vulnerable of all positions. I also understood him to say he had never used a matrix. Well, if I should say I had never used one, then I should not get up on this floor and discuss the matter, because I would think there was a chance to learn something. I think a matrix is sometimes invaluable.

Dr. Parker: I think the idea of contour fillings and preserving the inter-proximate space was fully talked over as early as 1855 or 1856. I think the dental reports will show it. I agree with Dr. Field that the cervical borders are the most difficult to handle.

Dr. Rix: I wish to take issue with the paper and also with the gentleman who opened the discussion on the paper in regard to the advocacy of contour fillings being of late date. I well remember when cohesive foil first came into notice that some very able dentists advocated contour filling so as to preserve the interproximate space, and if I am not mistaken Dr. Watling demonstrated that method before this association.

Dr. Watling: I think I did something of that kind.

Dr. Sweetnam: Dr. Rix has been asleep. I never said anything about contour fillings.

Dr. Loeffler: I listened to the paper very carefully; was very much pleased with it, but it seems to me we have been talking about two or three things at the same time. If I understood the doctor right, his paper was about where decay started at the point of contact. We cannot assume that decay does not start elsewhere. We must stick to the point of contact or have another paper.

